# Sympathetic activity contributes to the fMRI signal

**DOI:** 10.1038/s42003-019-0659-0

**Published:** 2019-11-18

**Authors:** Pinar Senay Özbay, Catie Chang, Dante Picchioni, Hendrik Mandelkow, Miranda Grace Chappel-Farley, Peter van Gelderen, Jacco Adrianus de Zwart, Jeff Duyn

**Affiliations:** 1 Advanced MRI Section, LFMI, NINDS, National Institutes of Health, Bethesda, MD USA; 2 Vanderbilt University, Nashville, TN USA; 3 University of California, Irvine, CA USA

**Keywords:** Neuro-vascular interactions, Neurophysiology, Non-REM sleep

## Abstract

The interpretation of functional magnetic resonance imaging (fMRI) studies of brain activity is often hampered by the presence of brain-wide signal variations that may arise from a variety of neuronal and non-neuronal sources. Recent work suggests a contribution from the sympathetic vascular innervation, which may affect the fMRI signal through its putative and poorly understood role in cerebral blood flow (CBF) regulation. By analyzing fMRI and (electro-) physiological signals concurrently acquired during sleep, we found that widespread fMRI signal changes often co-occur with electroencephalography (EEG) K-complexes, signatures of sub-cortical arousal, and episodic drops in finger skin vascular tone; phenomena that have been associated with intermittent sympathetic activity. These findings support the notion that the extrinsic sympathetic innervation of the cerebral vasculature contributes to CBF regulation and the fMRI signal. Accounting for this mechanism could help separate systemic from local signal contributions and improve interpretation of fMRI studies.

## Abstract (web summary)

Özbay et al. show the contribution of fluctuations in sympathetic activation on global fMRI signals in human brain during sleep. Such an inference is based on simultaneously acquiring and correlating EEG K-complexes and episodic drops in finger skin signatures with BOLD-fMRI changes during sleep.

## Introduction

Functional magnetic resonance imaging (fMRI) allows the study of brain function based on the blood oxygen level-dependent (BOLD) effect, whereby blood oxygenation changes associated with increases in blood flow are triggered in response to local neuronal activity through neurovascular coupling^1,2^. Based on this, spatial patterns of fMRI signal changes during behavioral tasks as well as during rest allow one to make inferences about the brain’s functional subdivision.

In addition to spatially specific features that may reflect local cortical computation, fMRI also has a spatially widespread (“global”) contribution that may originate from various sources, which need not be neuronal. For example, a strong association has been reported between fluctuations in cardiac rate and respiration depth and rate^3–5^ on one hand, and fMRI global signal (GS) changes on the other. However, mechanisms by which autonomic physiology and other factors affect GS remain incompletely understood, complicating the study of cortical neuronal activity with fMRI.

One mechanism by which autonomic physiology may affect cerebral blood flow (CBF)—and therefore the fMRI signal—is through changes in blood CO_2_ concentration, a potent cerebral vasodilator^6,7^. For example, in intracortical arterioles, an increase in CO_2_ concentration may lead to local vasodilation^8^ and increases in the fMRI signal. This potential mechanism is supported by studies identifying a strong correlation between end-tidal CO_2_ and the fMRI signal^5,9^.

Intriguingly, recent fMRI studies have also reported a correlation between fMRI GS and (finger) skin vascular tone^10–13^, which is under the control of the sympathetic nervous system rather than a local CO_2_-dependent mechanism. As both skin vasculature and central nervous system (CNS) arteries are innervated by the sympathetic nervous system, this suggests a potential alternative or additional mechanism underlying the fMRI GS. Specifically, the sympathetic innervation of the CNS involves most extra-parenchymal arteries, consistent with a potential widespread influence on CBF^1,2,14^ and a contribution to fMRI GS. Furthermore, sympathetic activity is known to be prevalent during changes in alertness and arousal state as judged from its close association with α transitions and K-complexes in the electroencephalography (EEG) signal^15,16^, conditions also favorable to result in variations in fMRI GS^17–19^. If confirmed, the role of the sympathetic activity in the fMRI GS has major implications for the interpretation of both task-evoked and resting-state fMRI.

Nevertheless, while a role of the sympathetic nervous system in the regulation of CBF has been demonstrated in animal models^20^, its importance in human has remained controversial^14,21,22^, and little evidence exists for a sympathetic contribution to the fMRI GS or CBF in human. To address this, we specifically focused on the nature of brain and physiological signal changes during EEG K-complexes. The latter constitute EEG detectable cortical evidence of sub-cortical arousals^23–25^ during sleep, and reflect periods of close interaction between the CNS and the autonomic nervous system. K-complexes have been closely associated with episodes of increased sympathetic activity^26^, and finger skin vascular tone^27^ derived from photoplethysmography (PPG)^15,16,28,29^. An association of K-complexes with joint changes in fMRI GS and PPG would therefore further affirm the putative role of sympathetic activity in the fMRI GS.

## Results

### Data summary

We analyzed a subset of data selected from 11 subjects who participated in an all-night sleep study^30^, in which fMRI and EEG were acquired concurrently with measures of systemic physiology. Heart rate (HR), finger skin vascular tone, and respiratory volume (RV) were derived from the PPG and chest belt signals.

### Main findings

**Fig. 1:**
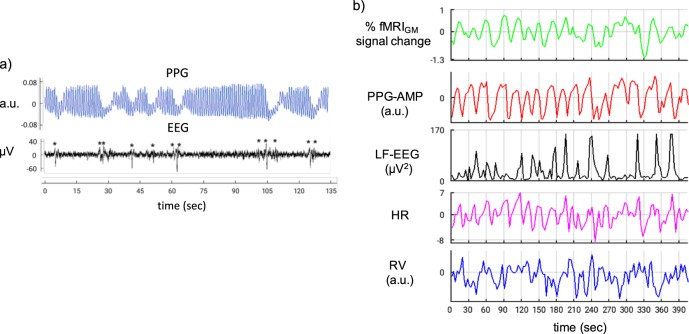
Covariation of fMRI, EEG, and autonomic signals during NREM2 sleep (subject S2). **a** Relationship between raw PPG and EEG (channel Fp1). K-complexes, indicated by asterisks, are typically followed by drops in the envelope of the PPG signal. **b** Relationship between fMRI and the various other signals. Episodic low-frequency (LF) EEG increases are apparently followed by drops in PPG-AMP and fMRI gray-matter (GM) signal. Relationship with variations in heart rate (HR, zero mean) and respiratory volume (RV) is less apparent. LF-EEG was calculated as the power in the 0.5–2 Hz spectral band for each 3 s (1 fMRI TR) interval, and clipped at two standard deviations for scaling purposes

Our analysis mainly focused on periods of moderate sleep depth (non-rapid eye movement stage 2, or NREM2), which tends to be punctuated by intermittent sub-cortical arousals visible in the EEG as K-complexes^25^. We first investigated the temporal relationship between variations in the amplitude of the PPG signal (PPG-AMP), and indicator of vascular tone, and the occurrence of EEG K-complexes. For this purpose, K-complexes were either identified from the EEG data automatically, or their temporal density was inferred from low-frequency EEG band-limited power (0.5–2 Hz, LF-EEG) (see Methods). PPG-AMP was highly variable during most of wake and NREM2 sleep, typically showing strong intermittent drops below an otherwise stable appearing baseline. Consistent with earlier work comparing PPG and EEG^15,29^, NREM2 was characterized by a strong temporal association between K-complexes and intermittent PPG-AMP dips (Fig. 1a). About 92% of detected K-complexes were associated with PPG-AMP drops (Supplementary Table 1). Typically, LF-EEG, PPG-AMP, and fMRI showed clear covariations, while the relationship with other autonomic indicators such as HR and RV being less obvious (Fig. 1b).

### Spatio-temporal cross-correlations

**Fig. 2:**
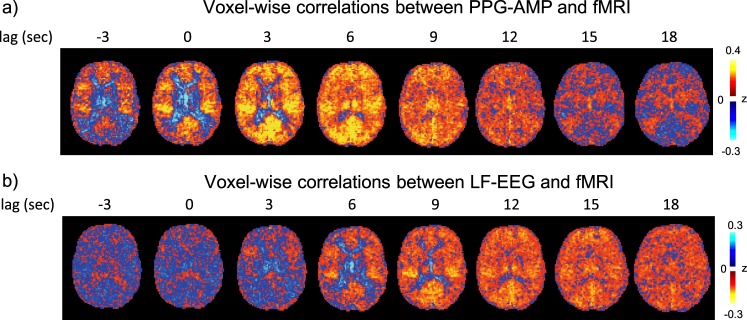
Relationship between fMRI and either PPG-AMP (**a**), or LF-EEG (**b**) during NREM2 sleep (*n* = 7). Single slice fMRI correlations are shown at various lags. Strong and spatially similar (anti-)correlation patterns are seen for both PPG and EEG, with the latter occurring at later lags. These observations are consistent with the notion that LF-EEG and PPG-AMP changes are strongly related, with the former preceding the latter. Note that that the color scale was inverted between the two comparisons to emphasize similarities. Correlations with LF-EEG are predominantly negative, consistent with a vasoconstrictive effect on cerebral arteries associated with sympathetic activity

The relationship between the various signals was further analyzed with cross-correlation analysis. First, we calculated the cross-correlation LF-EEG and PPG-AMP on one hand, and the voxel-level fMRI signal on the other. We observed widespread correlations, which were of similar magnitude and extent for both LF-EEG and PPG-AMP (Fig. 2, for thresholded maps see Supplementary Fig. 1). The PPG-AMP–fMRI correlation during wake was also highly similar to the pattern during light sleep (see Supplementary Fig. 2 for wake-state analysis). However, the LF-EEG–fMRI correlation showed opposite polarity and peaked at longer delays, suggesting EEG K-complexes precede both the drops in PPG-AMP and fMRI GS. We also observed a striking spatio-temporal pattern, with antipolar characteristics of the correlation between gray matter (GM) and peri-ventricular white matter.

**Fig. 3:**
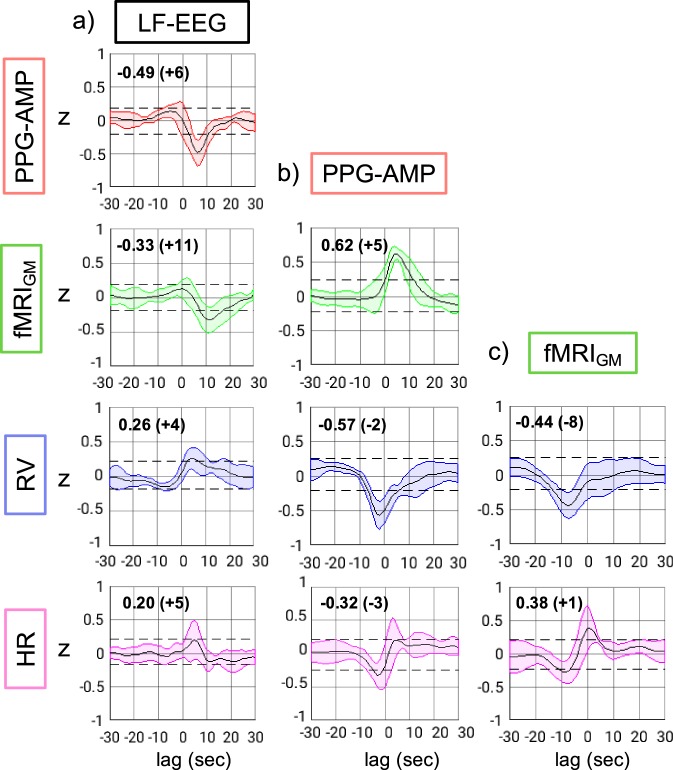
Lag-dependent cross-correlations between various signals, resulting from group level analysis (*n* = 7) of NREM2 sleep data. Cross-correlations which are above or below the dashed lines indicate statistical significance (*p* < 0.0125, corrected for multiple comparisons) against null hypothesis (see Methods). Strongest correlations of LF-EEG with all other signals occur at positive lags, indicating LF-EEG changes precede all other signals. Black lines are the mean temporal correlations of LF-EEG, PPG-AMP, and fMRI_GM_ with all other signals. Colored areas indicate standard deviations across subjects. Strongest correlations (mean Fisher’s *z*) with corresponding lags in seconds, within parentheses, are given on the upper left corner of each subfigure. As example, for LF-EEG vs. PPG-AMP, a negative *z*-score (*z* = −0.49) at a positive lag (lag = +6) indicates an increase in LF-EEG followed by a PPG-AMP drop

Cross-correlations between LF-EEG and all other physiological signals suggested that LF-EEG increases (attributed to K-complexes) each are followed by a significant drop in PPG-AMP (*z* = −0.49 ± 0.19, *p* < 0.01), and later by an fMRI signal decrease in GM (fMRI_GM_) (*z* = −0.33 ± 0.19, *p* < 0.01) (Fig. 3a). Similar results were obtained for signals averaged within brain regions associated with fMRI resting-state networks, for example, visual, motor, and default mode network (Supplementary Fig. 3). The fMRI_GM_ signal showed the strongest correlation with the PPG-AMP signal (*z* = 0.62 ± 0.08, *p* < 0.01) (Fig. 3b), while the correlation with RV (*z* = −0.44 ± 0.18, *p* < 0.01) and HR (*z* = 0.38 ± 0.27, *p* < 0.01) (Fig. 3c) was somewhat weaker. Strong correlation (*z* = −0.57 ± 0.20, *p* < 0.01) was also observed between PPG-AMP and RV.

### Event-locked analyses

**Fig. 4:**
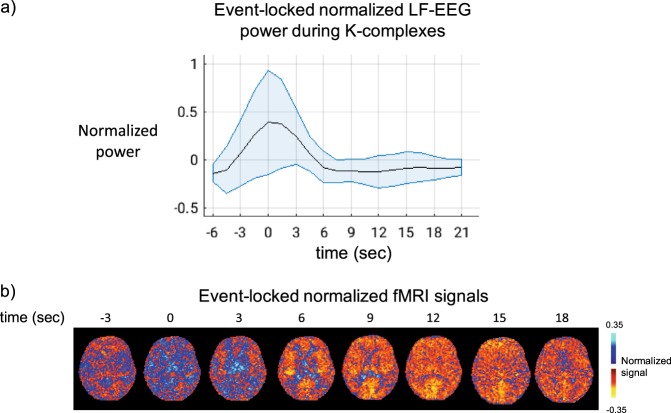
Confirmation of temporal relationship between K-complexes and fMRI with K-complex-aligned averaging of fMRI signals during NREM2 sleep. **a** Increased power around *t* = 0 s in the K-complex aligned average LF-EEG signal supports the use of LF-EEG as a surrogate for K-complexes. Black line and blue colored area give means and standard deviations (*n* = 7). **b** K-complex triggered average of fMRI signal shows pattern resembling that observed LF-EEG-fMRI correlation (Fig. 2b), suggesting that in much of gray matter K-complexes precede fMRI changes by about 6–12 s

To confirm the appropriateness of using LF-EEG as a proxy for K-complexes, we also inspected K-complex-triggered averages of the fMRI signals. This was performed by using timings of K-complex events derived from the output of the SLEEP toolbox (see Methods). As expected, this led to a result similar to that obtained by correlating LF-EEG with fMRI (Fig. 4), showing K-complexes preceding a fMRI signal drop involving most of the GM.

## Discussion

We examined concurrently acquired fMRI and physiological data for evidence of a potential sympathetic contribution to CNS vascular tone and fMRI GS. During NREM2 sleep, we found both the fMRI GS and finger skin vascular tone to be closely associated with the occurrence of EEG K-complexes, previously associated with episodic increases in sympathetic activity^30^. Prominent, albeit weaker correlation between K-complexes on one hand, and RV and HR changes on the other were also found. The observed joint changes in these signals, temporal precedence of K-complexes, and spatio-temporal pattern of associated fMRI signal changes are all suggestive of a systemic vascular contribution to the fMRI GS mediated by the extrinsic sympathetic innervation of the CNS vasculature. This so far overlooked contribution to the fMRI signal is distinct from contributions mediated by local (parenchymal) vascular mechanisms, including intravascular CO_2_ changes, action of the brain’s intrinsic innervation, or the vascular response to local neuronal activity. Since fluctuations in sympathetic activity are prevalent during most arousal states, with the exception of NREM3 (deep) sleep^31^, this effect is expected to contribute to GS under conditions typical of most fMRI experiments where it may confound interpretation. Inspection of NREM3 sleep segments from our dataset indeed showed reduced correlations between LF-EEG or PPG amplitude on one hand, and fMRI signal on the other (see Supplementary Fig. 4).

**Fig. 5:**
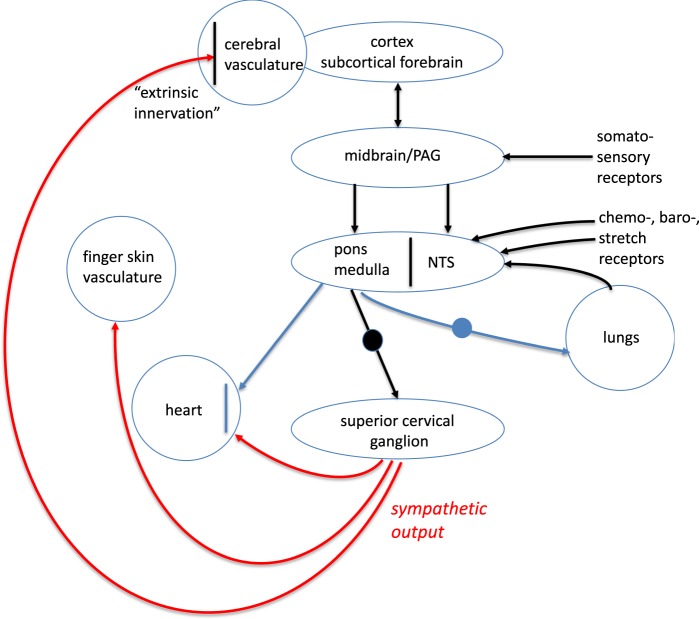
Schematic of arousal system showing sympathetic connections from superior cervical ganglion (SCG) to heart, peripheral, and CNS vessels, and to SCG from brainstem and input to nucleus tractus solitarius (NTS) from cortex and periphery. “Top-down” cortical input well as “bottom-up” receptor input to the brainstem can result in sympathetic output that simultaneously constricts vasculature in CNS and finger skin, resulting in joint changes in PPG-AMP and fMRI GS. In addition, pathways emanating from the brainstem exist that may effectuate coincident changes in heart rate and respiration. Freely adapted from ref. ^34^

During NREM2 sleep, a high coincidence rate between K-complexes and PPG-AMP dips was observed, with on average 92% of the K-complexes being associated with PPG-AMP dips (Supplementary Table 1). This phenomenon has been noted in EEG–PPG studies^15,16,32^ and is known as the “orienting reflex”^29^. Alertness and arousal changes during wake, or sub-cortical arousal during sleep may trigger episodic sympathetic vasoconstriction through an interaction between the neural substrates regulating arousal and the vascular baroreflex^33,34^. Such triggering may result from physiological stimuli (e.g., sensory, pressure, CO_2_, respiration) arriving through ascending pathways or cognitive psychological stimuli through descending pathways (Fig. 5, see review by Dampney^33^). Therefore, a sympathetic contribution to GS is also more generally expected with alertness changes during resting wakefulness that typically occur in resting-state fMRI experiments^35^, similar to sub-cortical arousal during light sleep stages as indexed by K-complexes. In fact, EEG α fluctuations have also been found to be associated with finger skin vasoconstriction^15,36^. Sympathetic vasoconstriction may also be elicited with various stimuli and changes in environment^15,37^, which may explain some of the GS reductions observed with task changes^38,39^.

The association of K-complexes with PPG-AMP and the fMRI signal may also point to a mechanistic explanation for previous reports of a link between fMRI GS and variations in HR and RV^4,40^. Specifically, widespread fMRI signal changes may result from a systemic response mediated by brainstem arousal through the extrinsic innervation of CNS arteries, rather than—or in addition to—a local (parenchymal) response to the blood pressure (BP) and blood CO_2_ accompanying changes in HR and respiration. In fact, arousal changes during K-complexes often coincide with HR and respiratory variations. We also observed inter-subject variability in cross-correlations of PPG-AMP and respiratory variations, which included cases with strong PPG-AMP drop without a clear RV increase (Supplementary Fig. 5). The temporal analyses performed in this study, as well as in a previous work^12^, suggest that K-complexes indeed precede the changes in systemic physiology and fMRI GS. Nevertheless, the causal relationship between changes in systemic physiology and brainstem arousal remains unclear and may depend on conditions, in part due to the close integration between the underlying substrates^34^. For example, voluntary changes in breathing rate or depth may trigger sympathetic activity as well, not involving an initiating arousal^41,42^.

As in previous work^10,12^, a strong correlation between PPG amplitude and fMRI was observed. This is consistent with the notion that CNS and finger skin vascular tone are regulated from the same region (the superior cervical ganglion) of the sympathetic nervous system, and CNS vascular tone is a major factor in the BOLD contrast mechanism. For this reason, the use of PPG-AMP as a physiological reference for fMRI studies may be complimentary to and have advantages over other physiological measures such as HR^5^ and respiration^3^. Nevertheless, the results presented here suggest that removal of GS from fMRI signal should be done with caution. In this regard, it is important to note that the effect of sympathetic regulation of CNS vascular tone may not be uniform across brain regions^10,11^, and that parasympathetic mechanisms may contribute as well^43^. Furthermore, sympathetic vascular tone is likely to have a co-modulating neuronal component, originating from modulatory neurotransmitter systems projecting widely to cortex from various nuclei that are associated with arousal, including locus coeruleus, basal forebrain, raphe, and fastigial nuclei^44,45^. In addition, sympathetic changes with K-complexes and EEG alpha transitions likely also have a cortical activity component. For K-complexes, the associated neuronal effect on the fMRI signal is likely to be small as they are temporally sparse, and the long delay of around 10 s between K-complexes and associated fMRI signal changes (Figs. 2–4) is inconsistent with the known delays of about 4–5 s for the fMRI hemodynamic response to neuronal activity^46^. Nevertheless, any cortical activity contribution to (sub)-cortical arousal signals, small as it may be, may also be removed by regressing out the PPG-AMP signal, and this may not be desirable.

The strong association between PPG-AMP and K-complexes on one hand, and fMRI on the other, confirms the notion that the sympathetic nervous system contributes to the (phasic) regulation of  CNS blood flow, which has been a controversial and poorly studied issue^14,22^. The positive correlation between the GM fMRI signal and PPG-AMP suggests that sympathetic activity has constrictive effects on both CNS and finger skin vasculature. This makes sense in light of strong sympathetic innervation of both the finger skin vasculature and of pial arteries, and the fact that this innervation originates from a common source, the superior cervical ganglion (Fig. 5). The opposing effects seen in peri-ventricular white matter have been explained previously and may be due to a temporal mismatch between blood volume and blood oxygenation changes in the deep medullary vasculature resulting from sympathetic vasomotion occurring remotely^12^.

A sympathetic contribution to CNS vascular tone may partly explain spatial activity patterns found in previous fMRI studies^11,47–50^. It may also explain previous MRI observations during sleep, most notably the discordant findings for K-complexes where both widespread increases^51,52^ and decreases in associated fMRI signal were observed^52,53^. Rather than, or in addition to, local reductions in neuronal activity, previously observed decreases may have resulted from purely vascular effect associated with sympathetic activity. In addition, the proposed sympathetic mechanism may explain previously observed fMRI signal decreases with external stimuli^54^, the increases in fMRI signal fluctuations during light sleep^17,55^, and, more generally, the variation of fMRI GS with sleep stage^50,56^. A sympathetic origin may also contribute to the previously reported widespread correlation with electrophysiological and behavioral indices of arousal^18,57–60^. This suggests that previous work correlating arousal state or related phenomena such as slow waves with fMRI should be interpreted with caution.

The fMRI correlate of K-complexes reported here resembles fMRI patterns associated with spontaneous pupil dilations^61^ and the skin conductance response^62^: this makes sense as these in part reflect sympathetic (de-)activation. It also resembles the fMRI patterns observed with alertness fluctuations as determined from EEG alpha power^18,58,59,63,64^ consistent with the established relationship between alertness and sympathetic activity and their partly overlapping neuro-anatomical substrates^65,66^.

The observed fMRI correlate of putative sympathetic activity during sleep broadly involves the cortex but is not spatially uniform. This in part may be related to non-uniformities in the sympathetic innervation^67–69^, vascular density, hemodynamics, and blood oxygenation variations, and possibly a co-modulation of neural activity that also contributes to the fMRI signal. For example, cholinergic, serotonergic, and adrenergic signaling associated with (sub-) cortical arousal may covary with sympathetic activity and modulate cortical activity. During wake and light sleep, “top-down” regulation of sympathetic activity may also be associated with substantial cortical activity and associated fMRI signal.

The results presented here suggest an important contribution of sympathetic vasoconstriction of the CNS vasculature in the global BOLD fMRI signal, and it is likely that this finding is not limited to sleep but also relevant under the sympathetic fluctuations that may occur during typical resting-state and task-based fMRI studies. Nevertheless, it should be realized that other factors may contribute to the fMRI GS as well, including large-scale changes in neural activity^57^, local CO_2_-mediated vasodilation associated with respiratory changes^70,71^, direct effects of BP variations on CBF, and direct neurogenic control of CBF through the intrinsic innervation arising from (neuro)modulatory centers such as basal forebrain, raphe nucleus, and locus coeruleus^72^. In fact, a recent study showed substantial ispilateral reduction in fMRI signal fluctuation with unilateral basal forebrain inactivation using muscimol injection in primate^73^. In addition, widespread fMRI signal *increases* have been associated with brief changes in BP elicited by electrical stimulation and vasoconstrictive drugs in rats and mice^20,74^. The latter seems inconsistent with the current finding of fMRI signal reductions with K-complexes, as K-complexes are known to be associated with BP increases^28,31^. A possible explanation is that in the current study, a stronger accompanying CNS vasoconstrictive response counteracts (and overwhelms) any positive effect of BP on CBF. An apparently lower CNS vasoconstrictive response with the previous studies may be attributable to species difference, anesthetics, and a lower level of sympathetic activation associated with their experimental protocols. Clearly, effects of autonomic physiology are important and have as of yet not adequately dealt with in fMRI studies. Proper accounting for these effects will likely require the monitoring of the full spectrum of available physiological parameters, including respiratory depth and rate, finger skin vascular tone, HR, blood pressure, and possibly others as well.

The temporal coincidence of K-complexes and transient drops in the PPG-AMP as well as the fMRI signal during sub-cortical arousal changes are indicative of a common, sympathetic mechanism contributing to the fMRI GS. This confirms earlier research suggesting a sympathetic role in the dynamic regulation of CBF and may explain a number of previously unexplained fMRI observations, such as large variations in fMRI GS during sleep and wakefulness^17–19^, and some of the GS reductions observed with task changes^38,39^. It also suggests that a proper interpretation of brain function and functional connectivity from fMRI signals should take this mechanism into account. This will require a comprehensive analysis of the interaction between various physiological parameters, including respiration, HR, blood pressure, and peripheral vascular tone.

## Methods

Data were selected from a recent all-night EEG-fMRI sleep study^30^. The selection was primarily based on previously established quality criteria^12^, including the absence of head motion (translation in excess of 2 mm, or rotation in excess of 2° judged from the fMRI time-series), and PPG signal with a clearly visible heartbeat. Here, we further limited data selection based on PPG data without finger motion artifact and nearly continuous NREM2 sleep (each lasting between 5 and 8 min). Subjects also underwent traditional resting-state scans before and/or after the sleep scans, and were asked to stay awake with eyes closed. We included six of these fMRI scans, each from one subject and lasting 5 min. In total, 21 segments from 21 fMRI scans, from *n* = 11 subjects (7 female, age range = 21–31), were analyzed. Of these 21 segments, 9 segments of sleep data (*n* = 7) consisted of 80–100% of NREM2 sleep (Supplementary Table 1), 6 segments of resting-state data (*n* = 6) consisted of 80–100% wake (Supplementary Table 2), and 6 segments of sleep data (*n* = 6) consisted of 100% of NREM3 sleep (Supplementary Table 3). We also included an EEG spectrum, sleep scores, and physiological data from one subject, to illustrate transitions between sleep states (Supplementary Fig. 6).

### BOLD fMRI and EEG data acquisition

BOLD fMRI data were obtained at 3 T (Skyra, Siemens Erlangen, Germany) with gradient-echo-EPI and with a 20-channel receive array. Acquisition parameters were: flip angle = 90°, repetition time (TR) = 3 s, echo time = 36 ms, voxel size = 2.5 × 2.5 × 2 mm^3^, slice gap = 0.5 mm, matrix size = 96 × 70 × 50, acceleration factor = 2. EEG and ECG were acquired concurrently with fMRI using a 64-channel recorder (Brain Products GmbH, Germany) synchronized to the MRI system clock using a 5 kHz acquisition rate. EEG caps featured 61-scalp electrodes, 2 electro-oculography electrodes, and 1 ECG electrode^30^. EEG channels were referenced to the frontal–central midline electrode.

### Acquisition and preprocessing of physiological signals

PPG and chest belt signals were recorded with a Biopac system (Biopac, Goleta, CA, USA, using TSD200-MRI and TSD221-MRI transducers, an MP 150 digitizer sampling at 1000 Hz, and AcqKnowledge software). The transducer recording the PPG signal was placed on the left index finger, making sure heartbeats were visible in its signal. MRI scanner triggers were co-recorded with the physiological signals to allow data synchronization. Vascular tone was inferred from PPG amplitude (PPG-AMP), as the peak-to-trough amplitude of each pulse wave from the PPG signal^12^. The variation in HR was computed by averaging the differences between pairs of adjacent heartbeats contained in the 6 s window around each trigger and dividing the result by 60 (beats per minute)^5^. As a measure of RV time-series, we calculated the standard deviation of the respiratory waveform on a sliding window of 6 s centered at each trigger^5^.

### Preprocessing of fMRI data

Preprocessing of fMRI data included motion correction by aligning successive image volumes in the time-series using six-parameter rigid body image registration. Correction of residual motion artifacts was performed by regressing out the motion parameters from the first registration step together with their first derivatives, which was followed by masking out voxels outside the brain. Slowly varying signal drifts were regressed out using polynomial functions, with the order of the polynomial equal to the segment duration (in s) divided by 150. Lastly, slice-timing correction was applied to compensate for the fact that slices were acquired sequentially in time. Each preprocessing step was performed with AFNI (analysis of functional neuroimage routines software^75^, https://afni.nimh.nih.gov/afni).

### EEG sleep scoring and K-complex detection

After correction of MRI gradient artifacts and cardio-ballistic artifacts in the EEG data, and band-pass filtering the EEG signals between 0.3 and 35 Hz, sleep stages were manually scored in 30 s epochs with the aid of BrainVision Analyzer software^76^ (see Supplementary Fig. 6a, b for an exemplary EEG spectrogram, and corresponding hypnogram). Using a publicly available software (SLEEP)^77^, K-complexes were detected automatically (Fig. 1a). This was done using all 61-scalp channels. Probability and amplitude parameter settings for this software were 0.5 and 0.4, respectively, based on experimentally manipulating the values on an independent dataset with manually scored K-complexes.

### Event-locked and correlation analysis

To study the relationship between EEG, fMRI, and PPG, we calculated lag-dependent correlations based on Pearson’s correlations (*r*). As a proxy for K-complex activity during NREM2 sleep^78^, we calculated low-frequency band-limited EEG power between 0.5 and 2 Hz (LF-EEG, pre-frontal left position, Fp1) for each 3 s fMRI volume. This was done using a Hanning-tapered Fourier transform. Pearson’s correlations of LF-EEG and PPG-AMP with fMRI signals were performed on a voxel-by-voxel basis at various lags. Since the sampling distribution of Pearson’s *r* is not normally distributed, subject level correlations (*r*) were converted to *z*-scores (*z*) using the Fisher’s *z*-transform, and were first averaged within subjects with multiple segments, and then across subjects (Fig. 1).

To perform region-of-interest basis analyses, we averaged the fMRI signals of voxels within a GM mask (fMRI_GM_) calculated with the Brain Extraction Tool within FSL^79,80^. Additional temporal correlation analyses were performed with LF-EEG, PPG-AMP, and physiological signals (HR, RV). Pearson’s *r* is converted to *z*-scores (*z*) using Fisher’s *z*-transform (Supplementary Fig. 5) prior to averaging.

The validity of using LF-EEG as a proxy for K-complexes to investigate their relationship with fMRI was confirmed by averaging the fMRI time fragments based on the temporal occurrence of K-complexes (i.e., created K-complex triggered average fMRI signals). K-complex timing was extracted from the output of the SLEEP toolbox, and LF-EEG and fMRI signals were normalized to zero mean and unit variance.

Analysis of slow-wave activity was carried out also for NREM3 segments (Supplementary Table 3)^81^. To perform voxelwise correlations with fMRI, low-frequency EEG power (0.5–2 Hz, Fp1)^25,82^ was calculated as previously described for the EEG power analyses (Supplementary Fig. 4).

### Correlation analyses based on structural parcellation

In order to investigate the temporal relations of PPG-AMP and LF-EEG with resting-state networks, we identified the visual, motor, and default mode regions of each individual participant. We used Freesurfer’s automatic parcellation methods based on anatomical T1 maps^83^. The output of the reconstruction includes the parcellation with the Desikan-Killiany Atlas^84^, with the Destrieux Atlas^85^, as well as a segmentation of the sub-cortical areas (nuclei). We selected visual, motor, and default mode network (DMN)-related volume of interests (VOIs) per hemisphere, which are the most common brain structures included in the resting-state studies. The VOIs included following labels of FreeSurfer: visual network included calcarine, cuneus, precuneus, parietal occipital, and temporal medial lingual; motor network included precentral inferior part, central sulcus, and gyrus; and DMN included medial orbitofrontal, rostral anterior cingulate, posterior cingulate, and posterior cingulate. We then aligned and resampled these VOIs with respect to the functional data with AFNI routines (3dAllinetate and 3dresample), multiplied with GM mask to avoid partial volume effects. We calculated temporal cross-correlations of LF-EEG and PPG-AMP with fMRI signal within the VOIs (Supplementary Fig. 3).

### Statistical analyses

To assess the statistical significance of the lagged cross-correlations we estimated a 99% confidence interval based on percentiles (corrected for multiple comparisons via Bonferroni correction, number of comparisons = 4) of an empirical null distribution (Fig. 3). The null distribution was sampled by computing and pooling all possible lagged cross-correlations for each of the vector pairs by circular shifting but excluding absolute lag times <30 TR.

We also performed a voxelwise statistical analysis: a null distribution for the spatial maximum absolute fMRI correlation with the PPG-AMP (or LF-EEG) was sampled by random shifting in time, as described above, and averaging each shifted correlation map across subjects before finding the spatial maximum. In line with the “voxel-based thresholding” option of “randomize” function of FSL^80,81^, we used the 95th percentile of this empirical null distribution of spatial maxima as a multiple-comparison-corrected significance threshold applied on the mean voxelwise correlation maps (see Supplementary Fig. 1).

### Reporting summary

Further information on research design is available in the Nature Research Reporting Summary linked to this article.

## Supplementary information

Supplementary Information

Descriptions of supplementary data

Supplementary Data 1

Supplementary Data 2

Supplementary Data 3

Supplementary Data 4

Supplementary Data 5

Supplementary Data 6

Supplementary Data 7

Supplementary Data 8

Supplementary Data 9

Supplementary Data 10

Reporting Summary

## Data Availability

The datasets generated or analyzed during the current study are available from the first or corresponding author on reasonable request. All source data underlying the main figures (Figs. 1–4) are available as Supplementary Data.

## Code availability

The scripts generated and used in the current study are available from the first or corresponding author on reasonable request.
