# Supplementary material for: Sympathetic activity contributes to the fMRI signal
Source: Commun Biol. 2019 Nov 18;2:421. doi: 10.1038/s42003-019-0659-0 (PMC6861267; doi:10.1038/s42003-019-0659-0)
Supplement: Supplementary file 1 — Supplementary Information [file 42003_2019_659_MOESM1_ESM.pdf]

Supplementary Information

Supplementary Table 1: Coincidence of PPG-AMP drops and K-complex in sleep data segments with predominantly NREM2 sleep selected from 7 subjects (S1 to S7).

Subjects	Length of segments (in TR)	Total number of Kcs	Kcs during dips	Number of dips	Dips without Kcs	Sleep stage (%)			
						NREM1	NREM2	W	NREM3
S1	150	14	11	8	1		100		
S2	115	40	40	20	0		100		
	130	41	34	13	0	7	93		
S3	160	10	10	14	2	19	81		
S4	150	37	36	25	0	7	93		
S5	120	21	21	18	3		100		
	110	19	19	23	3		100		
S6	120	14	14	17	2		100		
S7	102	25	20	10	1		80	10	10
Total		221	205	148	12				

NREM1-3: non-rapid eye movement sleep stages of 1, 2 and 3, W: wake.

Supplementary Table 2: Fraction of sleep stages in resting-state data used for wake-state analysis (n=6).

Subjects	Length of segments (in TR)	Sleep stage (%)	
		W	NREM1
S1	100	100	
S6	100	100	
S7	100	100	
S8	100	100	
S9	100	100	
S10	100	80	20

NREM1: non-rapid eye movement sleep stage of 1, W: wake.

Supplementary Table 3: Fraction of sleep stages used for NREM3 analysis (n=6).

Subjects	Length of segments (in TR)	Sleep stage (%)
		NREM3
S1	110	100
S2	100	100
S4	130	100
S5	200	100
S6	200	100
S11	140	100

NREM3: non-rapid eye movement sleep stage of 3.

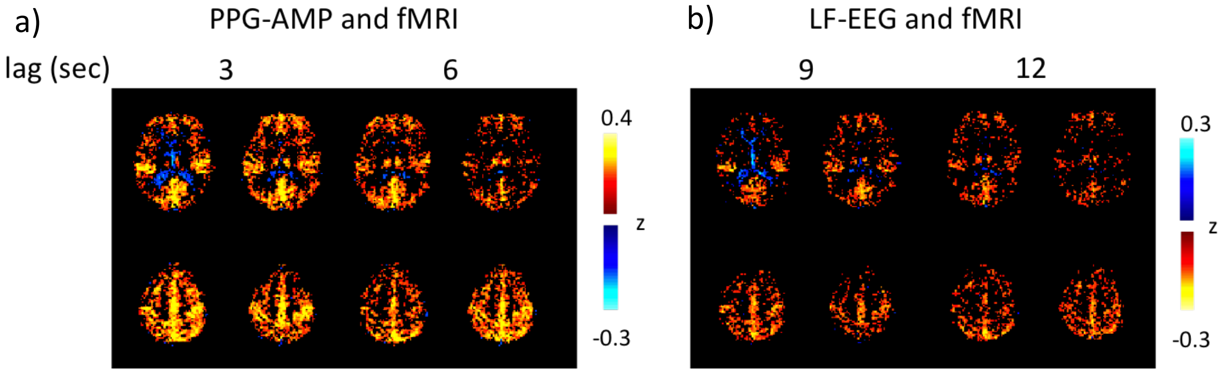

Supplementary Figure 1: Group level (a) PPG-AMP and fMRI, and (b) LF-EEG and fMRI significant voxel wise correlations ($p < 0.05$, corrected for multiple comparisons) during NREM2 sleep (n=7).

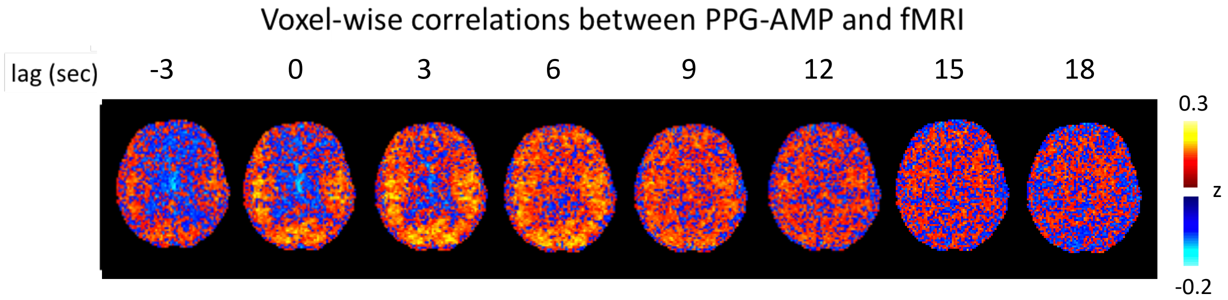

Supplementary Figure 2: Voxel-wise correlations between PPG-AMP and fMRI during wake segments (see **Supplementary Information Table 2**) of resting-state data (n=6) at various lags.

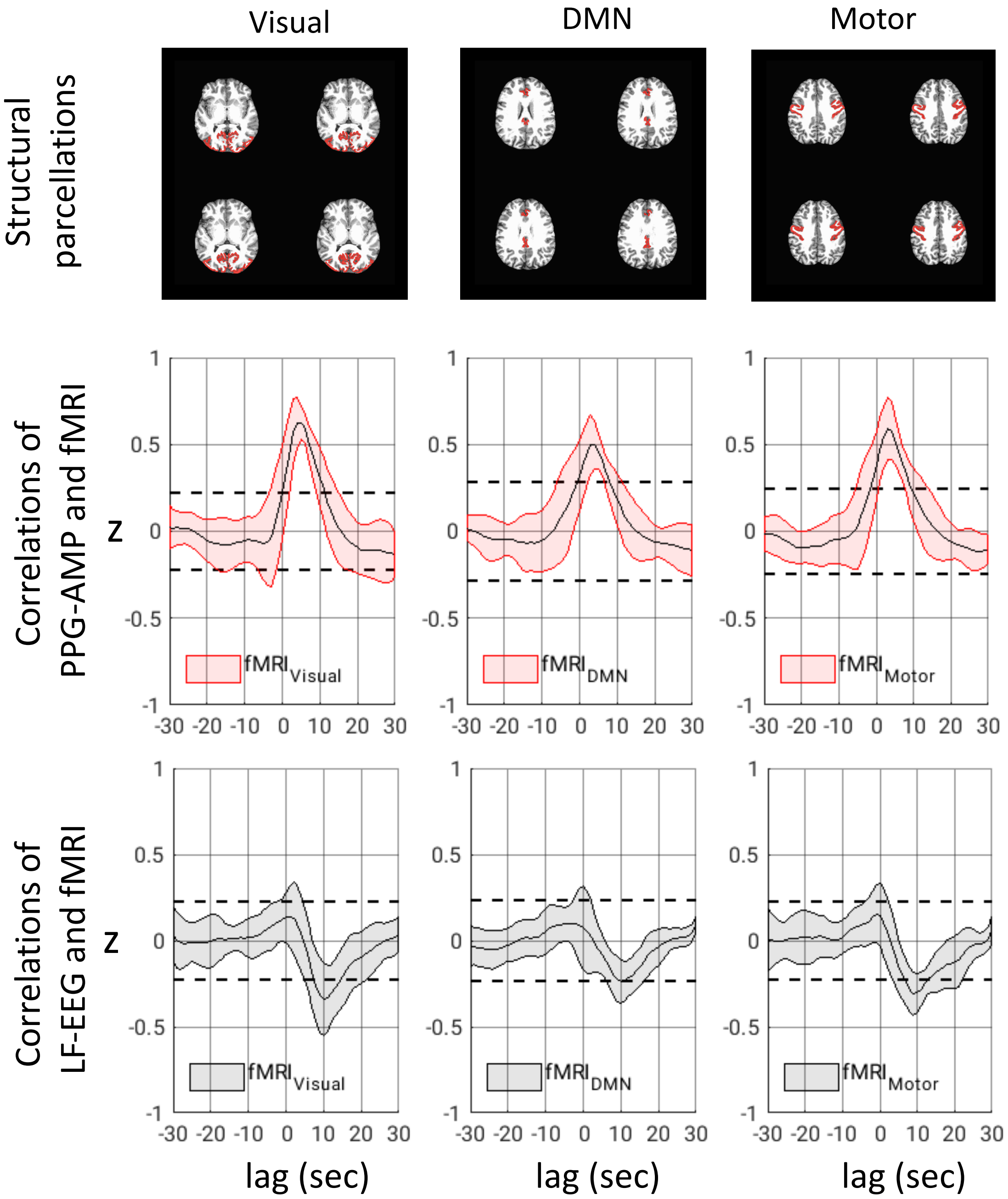

Supplementary Figure 3: Lagged cross correlations of PPG-AMP and LF-EEG with fMRI within selected networks during NREM2 sleep (see **Supplementary Table 2**) (n=7). Cross-correlations above the upper or below the lower dashed line are significant ($p < 0.025$, corrected for multiple comparisons).

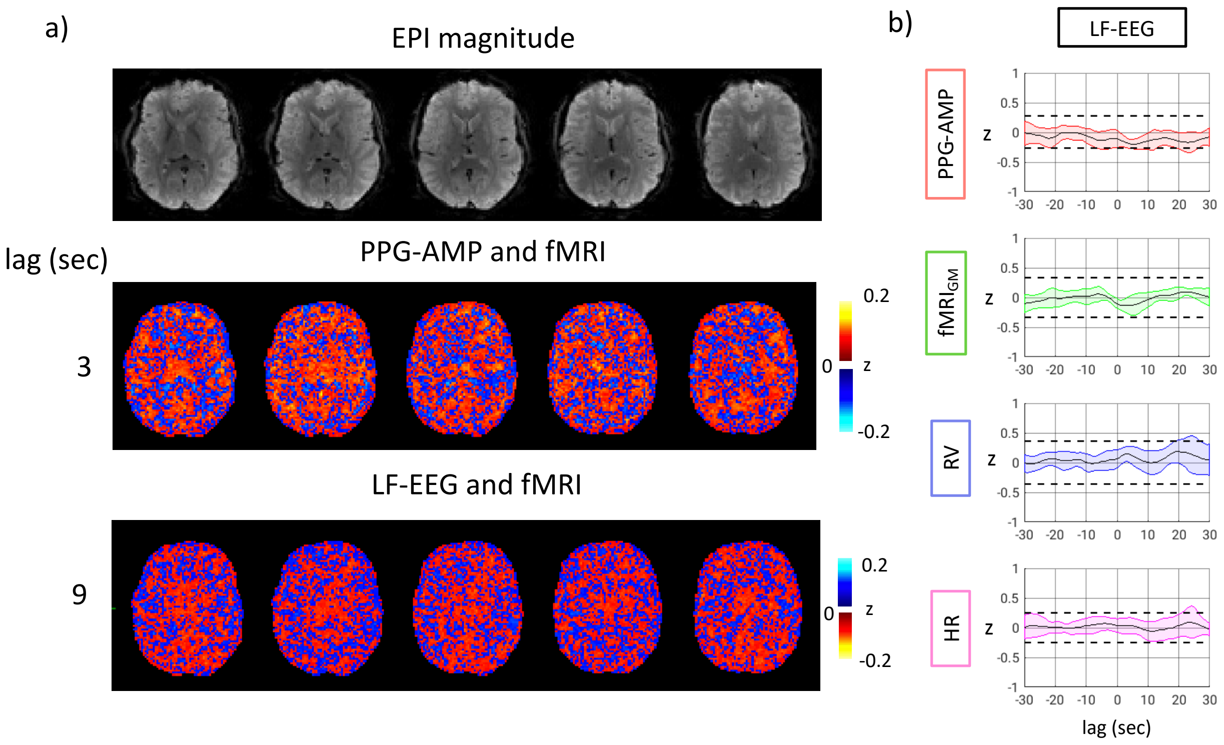

Supplementary Figure 4: (a) Contiguous slices of voxel-wise correlations of PPG-AMP and LF-EEG with fMRI during NREM3 sleep (see **Supplementary Table 3**) (n=6) at lags of 3 sec and 9 sec, respectively. (b) Lagged cross-correlation plots of LF-EEG with other signals. Correlations do not exceed significant levels ($p < 0.0125$, corrected for multiple comparisons), which are indicated by the horizontal dashed lines.

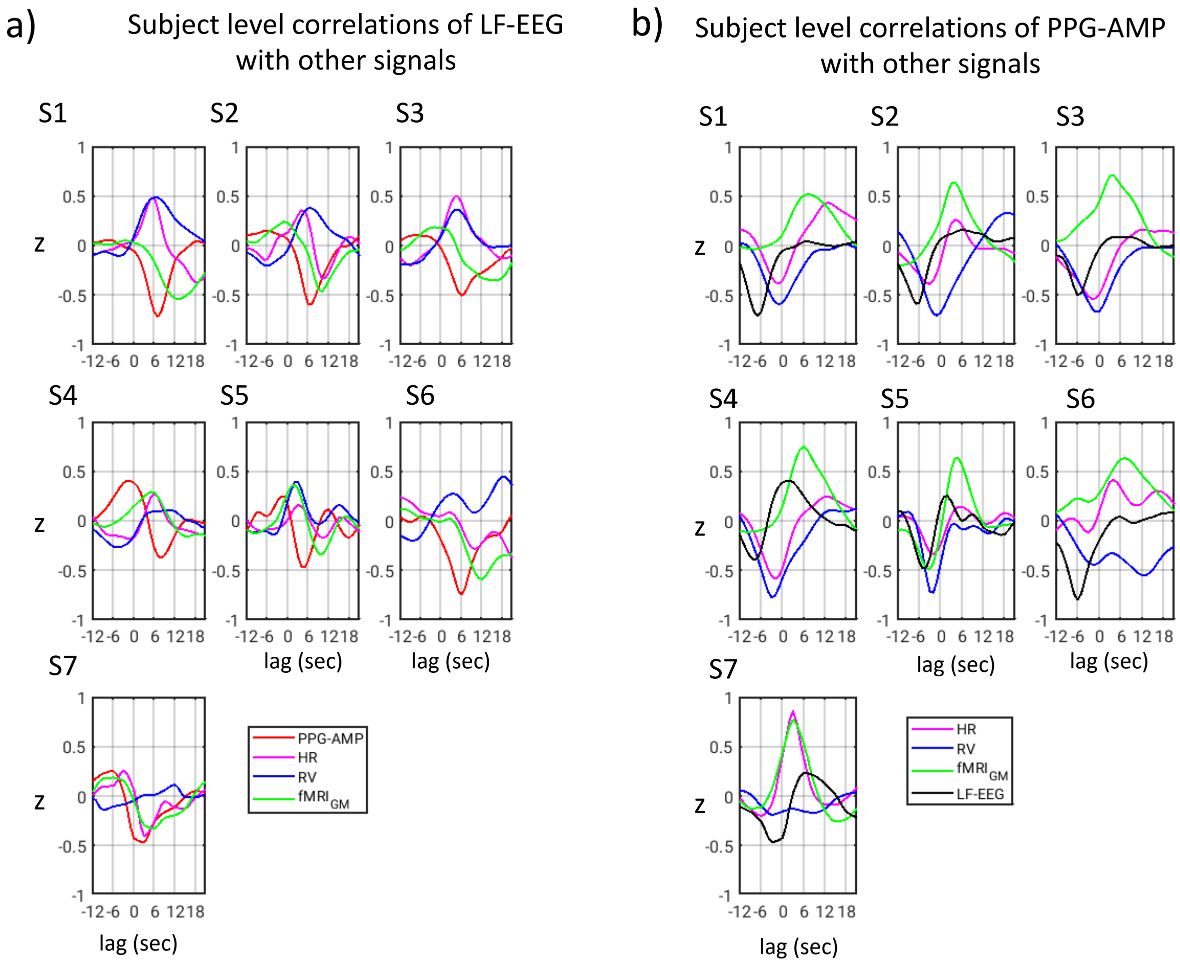

Supplementary Figure 5: Subject level cross-correlations (Fisher's z) between (a) LF-EEG and (b) PPG-AMP with other signals during NREM2 at various lags.

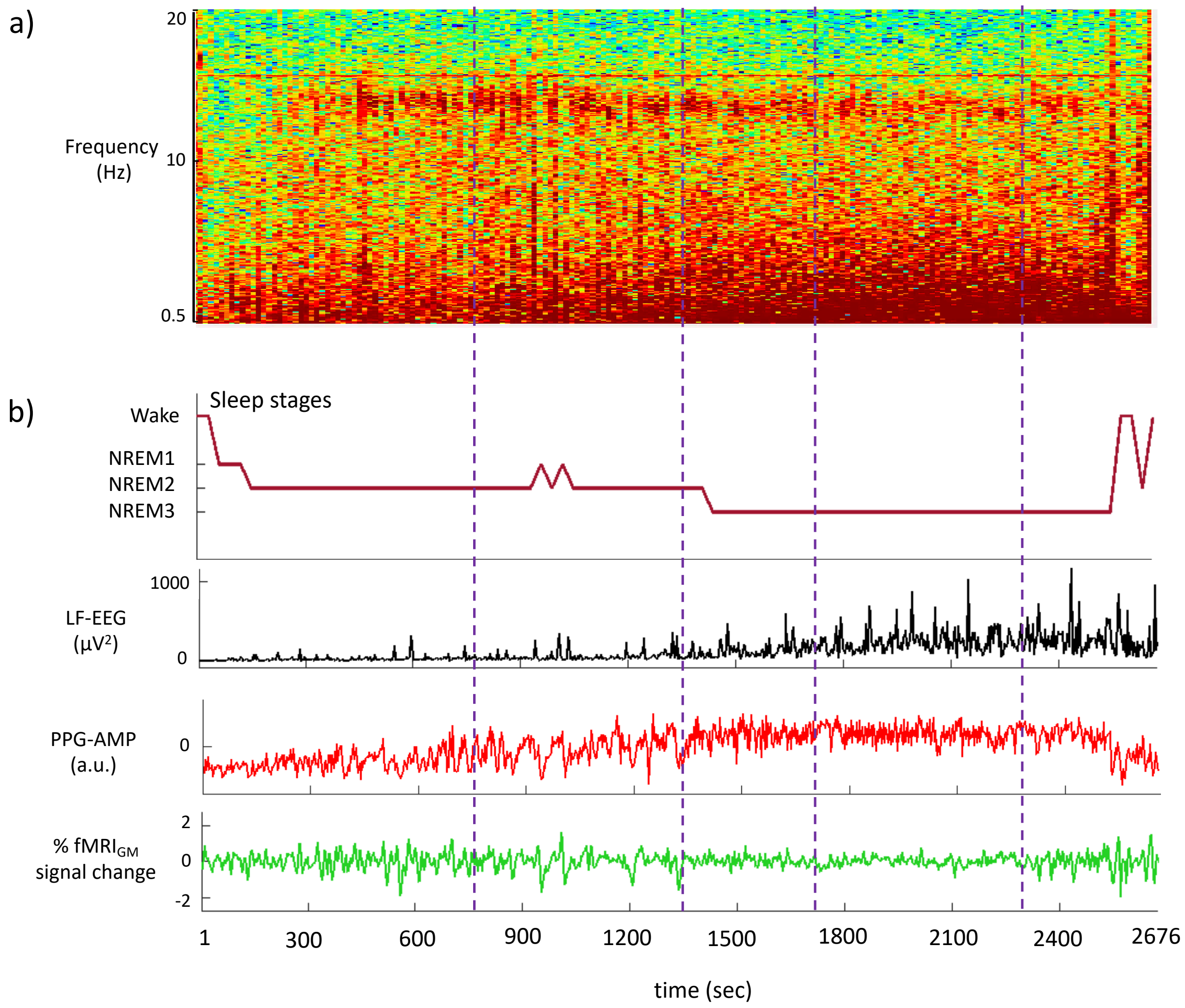

Supplementary Figure 6: Illustration of signal variations across recording segment including NREM2 and NREM3 sleep. (a) EEG spectrogram (Fp1) with warm colors indicating high power, and (b) corresponding sleep stages, time course of LF-EEG, PPG-AMP, and fMRI grey-matter ($fMRI_{GM}$). For visualization purposes, PPG-AMP spikes exceeding two standard deviations (attributed to finger movement) were clipped.
