# Supplementary material for: Sympathetic activity contributes to the fMRI signal
Source: Commun Biol. 2019 Nov 18;2:421. doi: 10.1038/s42003-019-0659-0 (PMC6861267; doi:10.1038/s42003-019-0659-0)
Supplement: Supplementary file 2 — Descriptions of supplementary data [file 42003_2019_659_MOESM2_ESM.docx]

Supplementary Data 1: Data related to Figure 1a; raw PPG signal from a single subject.

Supplementary Data 2: Data related to Figure 1a; raw EEG (Fp1) signal from a single subject.

Supplementary Data 3: Data related to Figure 1b; fMRI, PPG-AMP, LF-EEG, HR and RV temporal changes.

Supplementary Data 4: Data related to Figure 2a; averaged (n=7) voxel wise cross-correlations between fMRI and PPG-AMP from a single slice at various lags (each lagged correlation map is stored in one tab).

Supplementary Data 5: Data related to Figure 2b; averaged (n=7) voxel wise cross-correlations between fMRI LF-EEG from a single slice at various lags (each lagged correlation map is stored in one tab).

Supplementary Data 6: Data related to Figure3a, subject level lag-dependent cross-correlations between LF-EEG and PPG-AMP, fMRI, RV and HR signals (each cross-correlation is stored in one tab).

Supplementary Data 7: Data related to Figure3b, subject level lag-dependent cross-correlations between PPG-AMP and fMRI, RV and HR signals (each cross-correlation is stored in one tab).

Supplementary Data 8: Data related to Figure3c, subject level lag-dependent cross-correlations between fMRI and RV and HR signals (each cross-correlation is stored in one tab).

Supplementary Data 9: Data related to Figure4a, subject level event-locked normalized LF-EEG power during K-complexes (each subject data is stored in one tab).

Supplementary Data 10: Data related to Figure4b, event-locked normalized (K-complex triggered) average (n=7) of fMRI signals, from a single slice at various times (each time-lagged signal map is stored in one tab).
