# Supplementary material for: Sympathetic activity contributes to the fMRI signal
Source: Commun Biol. 2019 Nov 18;2:421. doi: 10.1038/s42003-019-0659-0 (PMC6861267; doi:10.1038/s42003-019-0659-0)
Supplement: Supplementary file 13 — Reporting Summary [file 42003_2019_659_MOESM13_ESM.pdf]

nature research

Corresponding author(s): Jeff H Duyn, COMMSBIO-19-0712B

Last updated by author(s): 10/10/2019

Reporting Summary

Nature Research wishes to improve the reproducibility of the work that we publish. This form provides structure for consistency and transparency in reporting. For further information on Nature Research policies, see Authors & Referees and the Editorial Policy Checklist.

Statistics

For all statistical analyses, confirm that the following items are present in the figure legend, table legend, main text, or Methods section.

n/a	Confirmed	
☐	☒	The exact sample size (*n*) for each experimental group/condition, given as a discrete number and unit of measurement
☒	☐	A statement on whether measurements were taken from distinct samples or whether the same sample was measured repeatedly
☐	☒	The statistical test(s) used AND whether they are one- or two-sided<br>*Only common tests should be described solely by name; describe more complex techniques in the Methods section.*
☒	☐	A description of all covariates tested
☐	☒	A description of any assumptions or corrections, such as tests of normality and adjustment for multiple comparisons
☐	☒	A full description of the statistical parameters including central tendency (e.g. means) or other basic estimates (e.g. regression coefficient) AND variation (e.g. standard deviation) or associated estimates of uncertainty (e.g. confidence intervals)
☐	☒	For null hypothesis testing, the test statistic (e.g. *F*, *t*, *r*) with confidence intervals, effect sizes, degrees of freedom and *P* value noted<br>*Give P values as exact values whenever suitable.*
☒	☐	For Bayesian analysis, information on the choice of priors and Markov chain Monte Carlo settings
☒	☐	For hierarchical and complex designs, identification of the appropriate level for tests and full reporting of outcomes
☐	☒	Estimates of effect sizes (e.g. Cohen's *d*, Pearson's *r*), indicating how they were calculated

Our web collection on statistics for biologists contains articles on many of the points above.

Software and code

Policy information about availability of computer code

Data collection	MRI: Siemens Skyra 3T scanner (Munich, Germany), Siemens IDEA VD13D, Gadgetron for MRI data reconstruction.<br>EEG: Brain products GmbH, Germany<br>Physiological data: Biopac, Goleta, CA, USA
Data analysis	Preprocessing of fMRI data was performed with AFNI routines, and EEG data with Brain Vision Analyzer software. Analyses were performed via custom MATLAB code.

For manuscripts utilizing custom algorithms or software that are central to the research but not yet described in published literature, software must be made available to editors/reviewers. We strongly encourage code deposition in a community repository (e.g. GitHub). See the Nature Research guidelines for submitting code & software for further information.

Data

Policy information about availability of data

All manuscripts must include a data availability statement. This statement should provide the following information, where applicable:

- Accession codes, unique identifiers, or web links for publicly available datasets
- A list of figures that have associated raw data
- A description of any restrictions on data availability

The datasets generated during and/or analyzed during the current study are available from the corresponding author on reasonable request.

Field-specific reporting

Please select the one below that is the best fit for your research. If you are not sure, read the appropriate sections before making your selection.

☒ Life sciences ☐ Behavioural & social sciences ☐ Ecological, evolutionary & environmental sciences

For a reference copy of the document with all sections, see nature.com/documents/nr-reporting-summary-flat.pdf

Life sciences study design

All studies must disclose on these points even when the disclosure is negative.

Sample size	n=11 subjects were used in the manuscript.
Data exclusions	We limited data selection based on PPG data without finger motion artifact and nearly continuous NREM2, NREM3 or wake state (each lasting between 5-8 minutes).
Replication	Not applicable.
Randomization	Not applicable.
Blinding	Not applicable.

Reporting for specific materials, systems and methods

We require information from authors about some types of materials, experimental systems and methods used in many studies. Here, indicate whether each material, system or method listed is relevant to your study. If you are not sure if a list item applies to your research, read the appropriate section before selecting a response.

Materials & experimental systems

n/a	Involved in the study	
☒	☐	Antibodies
☒	☐	Eukaryotic cell lines
☒	☐	Palaeontology
☒	☐	Animals and other organisms
☐	☒	Human research participants
☒	☐	Clinical data

Methods

n/a	Involved in the study	
☒	☐	ChIP-seq
☒	☐	Flow cytometry
☐	☒	MRI-based neuroimaging

Human research participants

Policy information about studies involving human research participants

Population characteristics	11 subjects (7 female, age range = 21-31)
Recruitment	Source of subjects: the NIH Clinical Research Volunteer Program registry, current NIH subjects, NIH staff, and individuals otherwise affiliated with NIH.<br>Recruitment venues: flyers were posted at NIH facilities. Notices were sent to Office of Patient Recruitment (list of those interested in receiving study recruitment updates) and NIH Listservs; posted on NIH Facebook pages, NIH Twitter accounts, Craigslist, and ResearchMatch; and published in NIH newsletters.<br>How potential subjects will be identified and approached: potential subjects self-identified. Advertisement placement venues: flyers were posted in conspicuous locations such as bulletin boards, and listserv notices will be sent to relevant listservs such as those comprised of postbaccalaureate fellows.
Ethics oversight	National Institutes of Health Combined Neuroscience Institutional Review Board (Protocol Number 16-N-0031).

Note that full information on the approval of the study protocol must also be provided in the manuscript.

Magnetic resonance imaging

Experimental design

Design type	Resting-state

Design specifications	The data used in the current work is from an all-night fMRI sleep study (Moehlman et al. 2019). In this work, the length of the segments used in the analyses was kept between 5-8 min.
Behavioral performance measures	No behavioral measures were used in the current study.

Acquisition

Imaging type(s)	fMRI
Field strength	3T
Sequence & imaging parameters	Gradient-echo-EPI, 3 Tesla. Acquisition parameters were: flip angle = 90 degree, repetition time (TR) = 3 s, echo time = 36 ms, voxel size = 2.5 x 2.5 x 2 mm3, slice-gap = 0.5 mm, matrix size = 96 x 70 x 50, acceleration factor = 2.
Area of acquisition	Whole brain scan

Diffusion MRI ☐ Used ☒ Not used

Preprocessing

Preprocessing software	Following preprocessing steps were performed with AFNI routines: motion correction, regressing out the motion parameters, masking out voxels outside the brain, regressing out slowly varying signal drifts using polynomial functions, and slice-timing correction (Analysis of Functional NeuroImages (AFNI) software75, https://afni.nimh.nih.gov/afni).
Normalization	All subjects data were registered to 1st subject's space.
Normalization template	No standard space was used in the current study, all data from the subjects were registered to 1st subject's space.
Noise and artifact removal	Motion correction by aligning successive image volumes in the time-series using six-parameter rigid body image registration, correction of residual motion artifacts was performed by regressing out the motion parameters from the first registration step together with their first derivatives, which was followed by masking out voxels outside the brain. Slowly varying signal drifts were regressed out using polynomial functions.
Volume censoring	Data selection criteria was based on the absence of head motion: translation in excess of 2 mm, or rotation in excess of 2° judged from the fMRI time-series.

Statistical modeling & inference

Model type and settings	N/A.
Effect(s) tested	N/A.

Specify type of analysis: ☐ Whole brain ☐ ROI-based ☒ Both

Anatomical location(s)	In addition to segmented whole brain grey-matter (via FSL, FAST), we used Freesurfer's automatic parcellation methods. The output includes the parcellation with the Desikan-Killiany Atlas, with the Destrieux Atlas, as well as a segmentation of the subcortical areas (nuclei). We selected visual, motor and default mode network (DMN) related regions for our analyses.
Statistic type for inference (See Eklund et al. 2016)	We assessed the statistical significance of the lagged cross-correlations by estimating a 99 % confidence interval based on percentiles of an empirical null distribution. We also performed voxel-wise statistics for correlation maps.
Correction	We included a voxel-wise statistical analysis based on a permutation test analogous to the 'voxel-based thresholding' option of the 'randomise' function in FSL (Smith and Nichols 2009; Jenkinson et al. 2012). Briefly, we use time-shifted correlations between the fMRI and PPG-AMP (or LF-EEG) to establish an empirical noise distribution for the maximum across space (voxels). In line with the 'voxel-based thresholding' (randomise, FSL), we used the 95th percentile of this empirical null distribution of spatial maxima as a multiple-comparison-corrected significance threshold. For temporal cross-correlation plots, we performed multiple comparison correction via Bonferroni correction.

Models & analysis

n/a	Involved in the study
☒	☐ Functional and/or effective connectivity
☒	☐ Graph analysis
☒	☐ Multivariate modeling or predictive analysis
